# More on Vasectomy

**Published:** 1911-01

**Authors:** G. Henri Bogart

**Affiliations:** Brookville, Indiana


### TEXAS MEDICAL JOURNAL.

Established July, 1885

F. E. DANIEL, M. D., - - - - - - Editor, Publisher and Proprietor

Published Monthly.—Subscription $1.00 a Year.

VOL. XXVI AUSTIN, JANUARY, 1911. No. 7

The publisher is not responsible for the views of the contributors.

### Original Articles.

For Texas Medical Journal.

### More on Vasectomy.

BY DR. G. HENRI BOGART, BROOKVILLE, INDIANA.

In the September number of the TEXAS MEDICAL JOURNAL, a paper of mine, discussing the various laws on the "Indiana plan" of preventing the procreation of degenerates, was published, and, in the December number a further paper was furnished. It now appears that there are progressive men who are leading a movement to have this great boon made a part of the law of Texas. With flattering prospects of success, and at the personal solicitation of Dr. F. E. Daniel, the editor, I am preparing the present paper.

There is an old song, which echoes the chorus—

"Tell it again,
Tell it again,
Salvation's story, repeat o'er, and o'er,"

and, in complying, I feel that this measure is one of the elements of the emancipation of humanity—material salvation.

A friend of mine, G. W. Miles, a State official, recently gave me an epigram: "There has never been anything accomplished by revolution but could have been better done by evolution," and evolution has for its keystone "The survival of the fittest," which correlatively includes the non-survival of the unfit.

"An ounce of prevention" was never better exemplified than in the safe, humane and simple prevention of the being of the greatest burden of humanity, through the workings of the law for vasectomy.

At the risk of tedium, I shall repeat the definition of this term and operation. There is a fibrous tube, the vas deferens, which carries the spermatozoa and orchitic fluid from the testicles to the vesicula seminalis, where the semen is fertilized.

In operating, the spermatic cord is held against the side of the scrotum, near the base of the penis, a slight incision is made and the seminal cord lifted up, the vas deferens is cut, and the vesical end ligated, to prevent the possibility of a subsequent gonorrhea running back; the testicular end left patulous, that the orchitic fluid may pour into the scrotal cavity, and be reabsorbed by the lymphatics, to the great benefit of the average subject, as this fluid is the "Brown-Sequard Elixir of Life."

Usually, no anesthetic is used, the operation is completed in three or four minutes, depending upon the facility of the surgeon.

There has never been any local congestion, nor other untoward result, and copulation, desire for sexual congress, ejaculation of semen, and all other concomitants of normal coition are practically unchanged.

These data are compiled from over six hundred cases in Indiana institutions, and nearly an equal number, reported to me from private practice, and extend back for twelve years.

I will say, in excuse for urging this question upon the readers of the "Red Back," that evolution is the development of the plans of the Omnipotent, but that every step must be accomplished by material means; that when the carter of classic story called upon Hercules to extricate his cart from the mire the god appeared, and commanded him to put his own shoulder to the wheel; while Cromwell's "Trust in God, and keep your powder 'dry," is proverbial.

The pioneer cleared away the primeval forest by cutting down one tree at a time, and we are chopping another chip into the evil of unfit procreation in this paper. That the degeneracy of the race is growing, far beyond the normal increase of the population, is too apparent to be questioned, as witness the quotation of the insanity expenses of the State of Texas in the editorials of the December number of this Journal.

Private cases include a hospital for syphilitics, in New York City, where many of the patients, before dismissal, after the matter has ben explained, take the operation, that they may avoid the horror of producing diseased offspring from those who have to do with excessive masturbators, etc.

In the latter case, even though the evil practice continue, there is no farther loss of the vital fluid. The most notable example of this latter line is reported by Dr. Carrington of the Virginia penitentiary, where ignorant negroes, shut in the solitude of their cells, give way to the vicious practice, and become maniacal, or, in less degree, neuresthenic, so that, though there is no procreation law yet on the statute books of the State, the surgeon is practicing the operation on this class as a remedial measure.

Sterilization by vasectomy is no longer an experiment, and is bound to come as the only possible remedy for the burden of the unfit.

Several States are represented by reform leaders who are working for this measure, educating the profession, as the first step; a fact with which, as a promoter of this gift to humanity, the writer has sure evidence from the immense correspondence on the question.

One of the notable papers on the extent and progress of degeneracy in America, is the inaugural address of Prof. Amos W. Butler, as President of the National Conference of Correction and Charities, at Minneapolis, in 1907, "The Burden of Feeblemindedness." A letter, inclosing postage, addressed to him at the State Board of Charities, Indianapolis, will bring to you this valuable paper.

He has followed a card index system of tabulating the feebleminded in Indiana, begun by E. P. Bicknell, reaching 3048 individuals; 57.3 per cent of these are children of degenerates, and possibly more, as there are many whose ancestry is unknown, while 1.4 per cent are the children of normal parents, who were within the prohibited limit of consanguinity; 13.8 per cent were illegitimate, which shows the futility of attempting to control evilbreeding, solely by marriage laws, as the Indiana law on marriage is considered one of the best.

It will lie with the medical man to master this operation, and to convey to the layman its plan and purpose, if we are to secure its benefits as law.

The argument, that the knowledge will be used criminally, is the veriest bosh. Already, for four decades, the similar operation on the famale has been extensively performed, illegally, on the well-to-do young women, who desired the pleasures of matrimony, without consequent maternity, and, in this illicit work, has been sapping the best blood of the country.

Few physicians but have already a knowledge of vasectomy, and, as I alone have furnished about two and a half articles per month for more than two years to medical journals from ocean to ocean, and abroad, and have furnished data to many doctors, and lectures of economics, it is too late to talk of keeping the operation from the knowledge of the masses.

Being one of the simplest and safest operations known to surgery, and coupled with the fact that the man operated upon gets out of the chair, and walks about his business, without even temporary inconvenience, makes it certain that it will be done more, and more commonly, and the only means of securing racial benefits from it lies in society taking it up in self-protection.

There is a question as to the constitutionality of sterilizing a man, and the answer is that a man has no more right to create a degenerate, who shall curse society and be a horror to himself, than he has to encroach upon social order in any other manner.

This question of "License vs. Liberty" has been used as the cloak behind which all varieties of iniquities have sough to hide themselves. There is another line from which consideration should be had, and that is Christian duty.

Christ made it a foundation stone of his ethical code that we are the keepers of our brothers. He defined sins of omission by saying, "Whoso is not for us is against us."

We talk learnedly of the right to be well born, and then forget the converse of allowing ill-birth. It is not to society alone that the burden falls. The crime of the career of a defective, a curse to himself, belongs justly to those who allowed his birth, could it have been prevented. Any legislator who, through ignorance, or sentiment, refuses to help pass a comprehensive law to prevent the conception of these creatures—the idiots, the epileptics, the natal criminals, and the like—may, in a few years, walk the streets, and, when one of them horrifies his sensibilities, congratulate himself that he directly helped to produce the pitiable object.

Texas has taken an advanced place as a State which does advanced things, and does them well, and I hereby appeal to the profession first, and through them to the broad intelligence of the citizens and lawmakers of the Lone Star State, to place the best procreation law of the world on their statute books.

---

Passing a catheter often cures post-operative abdominal pain not relieved by other means.—*American Journal of Surgery.*

---

Perforation may be the first serious sign of carcinoma of the pylorus as well as of ulcer.—*American Journal of Surgery.*

